# Pre-exercise meal on oxidation of energy substrates during maximal exercise test in non-trained individuals

**DOI:** 10.20945/2359-3997000000618

**Published:** 2023-05-29

**Authors:** Lucas Ribeiro da Silva, Giuseppe Potrick Stefani, Gilson Pires Dorneles, Aline Marcadenti, Pedro Dal Lago

**Affiliations:** 1 Universidade Federal de Ciências da Saúde de Porto Alegre Laboratório de Fisiologia Experimental Porto Alegre RS Brasil Laboratório de Fisiologia Experimental, Universidade Federal de Ciências da Saúde de Porto Alegre (UFCSPA), Porto Alegre, RS, Brasil; 2 Hospital do Coração Instituto de Pesquisas São Paulo SP Brasil Instituto de Pesquisas, Hospital do Coração (IP-HCor), São Paulo, SP, Brasil; 3 Universidade Federal de Ciências da Saúde de Porto Alegre Programa de Pós-graduação em Ciências da Reabilitação Porto Alegre RS Brasil Programa de Pós-graduação em Ciências da Reabilitação, Universidade Federal de Ciências da Saúde de Porto Alegre (UFCSPA), Porto Alegre, RS, Brasil; 4 Universidade Federal de Ciências da Saúde de Porto Alegre Laboratório de Imunologia Celular e Molecular Porto Alegre RS Brasil Laboratório de Imunologia Celular e Molecular, Universidade Federal de Ciências da Saúde de Porto Alegre, Porto Alegre, RS, Brasil

**Keywords:** Dietary fats, dietary carbohydrates, calorimetry, energy metabolism, exercise

## Abstract

**Objective:**

This study aimed to compare the influence of a high carbohydrate meal versus high-fat meal on the oxidation of substrates during an exercise incremental test.

**Materials and methods:**

Ten untrained male subjects underwent two days of the protocol. Randomly, they received a high carbohydrate meal or a high-fat meal, receiving the other one in the next protocol. On both days, they performed an incremental treadmill test, with heart rate and maximal oxygen consumption to estimate the oxidation of substrates.

**Results:**

The high-fat meal showed an increase in the absolute amount of oxidized fat along with the incremental test (*P* < 0.05; effect size = 0.9528), and a reduction in the respiratory exchange ratio at low intensities (*P* < 0.05; effect size = 0.7765).

**Conclusions:**

The meals presented no difference when compared to maximum oxidation point of substrates, the oxidation rate of substrates over time, and heart rate. A pre-test high-fat meal in untrained individuals was shown to be a modulating factor of total oxidized fats throughout the exercise, although it did not exert a significant effect on the rate of this oxidation over time.

## INTRODUCTION

Physical exercise, when practiced by a long time, is a trigger for adaptations at the skeletal muscle level, responsible for greater fat oxidation during exercise (1). Two main factors highlighted are the increase in intracellular fat deposits and in the number of mitochondria (2). Another relevant adaptation is the increase in the number of fatty acid transport proteins in such muscles, such as fatty acid binding protein and fatty acid translocase (3).

As an acute response to aerobic exercise, it is possible to observe a gradual suppression of lipid oxidation concomitantly as the exercise intensity increases (4). Such reduction in oxidation rate may be due to both the low availability of plasma fatty acids (reduction in lipolysis) and the inability of fat uptake and use by peripheral muscles as energy fuel (5).

Both situations can be explained by metabolic changes caused by the exercise. An increase in the intensity can stimulate catecholamines secretion, which can be observed in plasma. The effect of releasing higher catecholamines as exercise intensity rises is a reduction in blood flow in adipose tissue, thus reducing the capacity of distribution of the fatty acids (6). This hypothesis corroborates that the non-esterified plasma fatty acids are in reduced amount at high exercise intensities even though the rates of peripheral lipolysis are unchanged (7,8). However, the inability of mitochondrial fatty acid uptake in the peripheral muscles may be related to an exercise-mediated reduction of free carnitine (9), or a suppression of the activity of the enzyme carnitine palmitoyl transferase I, which can be explained by acidic micro-environment (10).

Although exercise intensity is the main determinant of fat oxidation, feeding can also modulate the concentration of substrates (5,6). A supply of carbohydrates, for example, can suppress the amount of free fatty acids, while fasting can increase them. Additionally, the level of training adaptation can directly influence the ability to oxidize fats during physical exercise. There is strong literature about the process of weight loss and fat oxidation, as mentioned above, however, the influence of acute dietary status on the further oxidation substrates, but in untrained individuals, the research it is still scarce (5,11). Thus far, it is not known if individuals who do not have chronic adaptations of aerobic training may be able to benefit from oxidizing more fats during physical activity with high-fat pre-exercise meals.

Due to this scenario, the present work aims to evaluate the influence of the macronutrient composition of a meal on the lipid oxidation curve in exercise. We used an incremental test to bring homogeneity to the intensities applied and consequently to the metabolic responses of individuals. The expected hypothesis is that a high-fat meal, when compared to a high carbohydrate meal, increases both the Maximum Fat Oxidation point (MFO) and the intensity at which maximal fat oxidation is observed.

## MATERIALS AND METHODS

### Ethical declaration

This study was conducted according to the guidelines laid down in the Declaration of Helsinki and all procedures involving human subjects were approved by the Ethic’s Research Committee of *Universidade Federal de Ciências da Saúde de Porto Alegre* (UFCSPA) and *Centro Universitário Metodista* (IPA), under Certificate of Presentation for Ethical Appreciation (CAAE) number 50272515.7.3001.5345. Written informed consent was obtained from all subjects.

### Study design

This is a controlled, cross over, and counter-balanced trial. Ten male participants performed two protocols with meals of different nutritional compositions followed by a motorized treadmill test in a randomized order with an interval of seven days between each experiment. All evaluations occurred in the morning shift.

**Figure 1:**
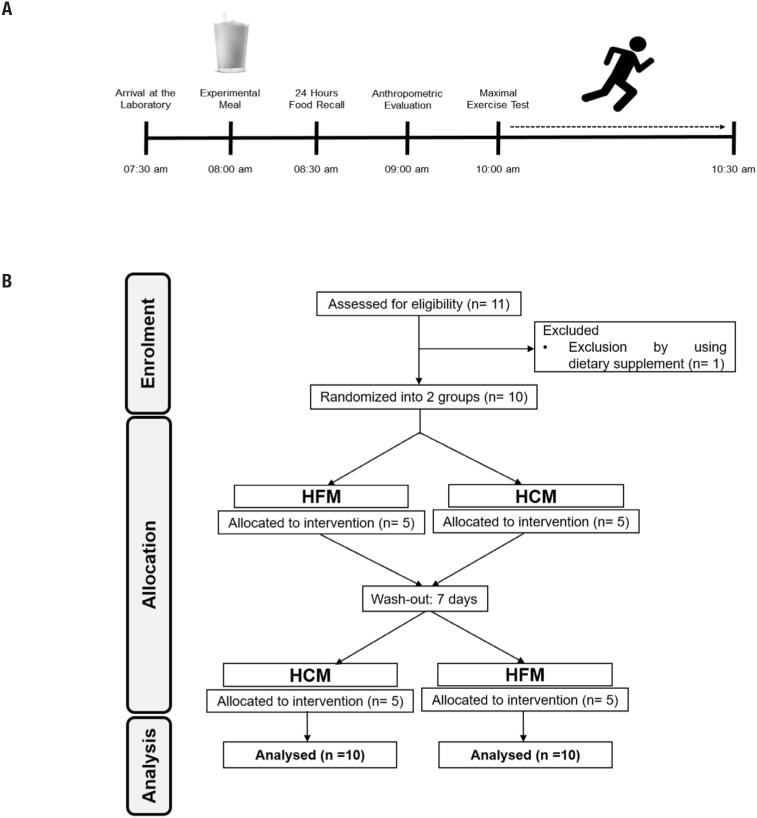
(**A**) Experimental timeline of the study. (**B**) Flow diagram of the study.

Each protocol lasted approximately three hours. According to Figure 1, the subjects arrived at the laboratory in fasting from 8 to 10 hours and ingested the test meal. During the next two hours, the volunteers remained at rest, so that all individuals maintained the same metabolic state until the beginning of the test. During this period, anthropometric assessments and 24-hour food recalls were done. Then the exercise test was performed. After one week, the same protocol was performed again, the only difference being the nutritional composition of the test meal and the absence of anthropometric evaluation.

### Participants and sample size calculation

To detect a minimum difference of 0.13 g/min and a standard deviation of 0.06 for maximum fat oxidation point as the main endpoint, with 95% power and error α = 5%, in a total of 2 experimental situations, the minimum number of participants for the study were 5 subjects (11). Sigmaplot software version 12.0 for Windows (San Jose, California, USA) was used for the sample calculation. A paired t-test was used for the sample calculation.

For inclusion criteria, the subjects were healthy, nonsmokers, reported non-habitual physical activity (less than 3 hours of exercise per week), and not taking any medications or supplements. Exclusion criteria included autoimmune, cardiac, endocrine, chronic conditions, or metabolic diseases, acute and chronic infections that would impact study outcomes. Volunteers were asked to refrain from strenuous physical activity for 48 hours before the protocols.

### Anthropometric evaluation

All measurements were performed in triplicates and the median was used for analysis. Total body mass (kg) and height (meters) were determined by a semi-analytical scale (Welmy, Santa Barbara D’Oeste, Brazil), with capacity for 200 kg and a stadiometer attached (Welmy, Santa Barbara D’Oeste, Brazil) with an accuracy of 0.1 kg and 0.005 cm, respectively. Waist (WP, cm), and hip (HP, cm) perimeters were measured through an inelastic measuring tape (Cescorf, Porto Alegre, Brazil). Body density and body fat percent were determined by the protocol of Guedes and Siri (12,13). All measurements of skinfolds were made on the right side of the body by using a compass (Cescorf, Porto Alegre, Brazil) with an accuracy of 0.1 mm. The same researcher performed all anthropometric evaluations in all participants.

### Food recall

Individuals were instructed to avoid the consumption of caffeine or alcoholic drinks for 24 hours before the protocols that would be followed. To clarify possible interference, they answered a food recall according to their meals in the 24 hours preceding the protocol in both days. The answers were evaluated in Dietbox Software (Porto Alegre, Brazil). The results are expressed in median and interquartile range.

### Composition of experimental meals

Experimental meals from this study were standardized for all volunteers containing approximately 380 kcal (isocaloric meal). All foods were weighed and consumed immediately. High Carbohydrate Meal (HCM) was composed of 72% carbohydrate, 13% protein, 15% fat, and 6.8 g of total fiber. The HCM consisted of semi-skimmed milk, oatmeal, banana, and honey. The HCM was prepared as a fruit shake (14). The High-Fat Meal (HFM) was composed of 21% carbohydrate, 9% protein, 70% fat, and 5.3 g of total fiber. The HFM consisted of natural yogurt, avocado, Brazil nut, and banana. The HFM was prepared as a mousse (15).

### Substrate oxidation rate

Average O_2_ uptake and CO_2_ production values during the last 2 minutes of each stage were used for further calculations. Whole-body carbohydrate and fat oxidation were calculated in each stage by using stoichiometric equations and appropriate energy equivalents. This equation considers that the urinary nitrogen excretion was negligible (16):


$$\begin{array}{l} \text{Carbohydrate Oxidation Rate}\;(\text{mg}/\min)=4.55\\ 3.21\times {\dot{\text{V}}\text{O}}_{2}(\text{mL}/\min) \end{array}$$

$$\text{Fat Oxidation Rate}(\text{mg}/\min)=1.67\times {\dot{\text{V}}\text{O}}_{2}(\;\text{mL}/\min)$$

The substrate oxidation rates were analyzed over a wide range of exercise intensities, expressed as a percentage of maximal oxygen uptake ( ${\dot{\text{V}}\text{O}}_{2\max}$ ). The resulting graphs were used to determine the maximal fat oxidation (MFO) rate (g/min), absolute consumption of substrates, and the exercise intensity at which the highest rate of fat oxidation was observed (Fatmax) for each individual through visual inspection by two independent evaluators.

The tests were divided into quartiles of intensity for graphical analysis. In this way, the tendency of the behavior of the studied variables could be established more easily, besides providing greater clarity of the metabolic ranges in which the individuals were during the test. To obtain the absolute amount of each oxidized substrate, we used a sum of all the oxidation points of the individual substrates, which were determined by the gas analyzer during the test. This data was summarized in a column chart.

### Graded incremental exercise test

The incremental exercise test was performed on an electric treadmill (Centurion 300, Micromed, Brasília, Brazil) using a graded exercise. We used this protocol for the ability to standardize the test. Because this protocol is well established and not individualized, homogeneity of effort (maximum) and data obtained from the stages between individuals is guaranteed. To ensure the same experimental conditions, the laboratory temperature and relative humidity were continuously monitored. The treadmill maximal exercise test protocol used in this study was described elsewhere (17). Briefly, the participants started exercising at a speed of 6.0 km/h and a 1.0 % grade for 3 minutes. The speed increased by 1.2 km/h and 1.0 % of inclination every 3 minutes until an RER (Respiratory Exchange Ratio) of 1.1 was reached. The test was ended by (a) request of the participant; (b) heart rate (HR) > 10 beats/min of the age-predicted maximum (HRmax), and (c) RER value > 1.1 (18). It should be noted that 3-minute stages were as effective as 5-minute stages in these graded tests

During each test, ventilation (VE), maximal oxygen consumption ( ${\dot{\text{V}}\text{O}}_{2\max}$ ), the release of carbon dioxide ( ${\dot{\text{V}}\text{CO}}_{2}$ ), ventilatory equivalents ( ${\text{VE}/\dot{\text{V}}\text{O}}_{2}$ and ${\text{VE/}\dot{\text{V}}\text{CO}}_{2}$ ) and respiratory coefficient (RER) were recorded. Ventilatory and metabolic parameters were collected breath-by-breath using Metalyzer (Cortex, Leipzig, Germany) and were analyzed after the mean of the data in 8 respiratory cycles in the Metasoft program (Medgraphics Cardiorespiratory Diagnostic Systems, model MGC/CPX-D, United States). The system pressure, volume, and gas analyzers were calibrated using a 3.0 L calibration pump and calibration gas (15.12% O_2_, 5.10% CO_2_). The heart rate was monitored during all exercise tests by a frequency meter (FT7, Polar, Finland) and the subjective perception of effort through the Borg Subjective Perceptual Scale (19).

### Statistical analysis

All data are presented in mean ± standard deviation (SD). To assess the normality of the variables included in the study, it was performed D’Agostino-Pearson. To compare different experimental meals, Student’s paired t-test was used. It was considered a significance level of 5 %. To further examine the statistical difference obtained, we performed Cohen’s d test to measure Effect Size test, followed by a power analysis of the comparison (20). We considered a reliable power analysis with at least 80.0%. All statistical analyses were performed in Sigma plot software version 12.0 for Windows (San Jose, California, USA), GraphPad Prism software version 7.0 for Windows (San Diego, California, USA), and GPower software version 3.1.9.2 for Windows (University of Düsseldorf, Düsseldorf, Germany). All images and graphs were created in GraphPad Prism software version 7.0 for Windows (San Diego, California, USA).

## RESULTS

The participants of this investigation were composed by men of mean age of 23.3 ± 3.6 years old. The sample showed slightly above eutrophic BMI with mean of 25.6 ± 3.2 kg/m². The body composition of untrained men showed to be a considerably high-fat percentage of 21.8 ± 4.2 %. The mean ${\dot{\text{V}}\text{O}}_{2\max}$ of the subjects was 42.8 ± 3.2 ml/kg/min. Regarding the 24 hours food recall, there was no difference between all of the variables of macronutrients and energy when comparing the two experimental situations. This result reinforces that all different meals did not influence of the day before the experiment. All baseline information is presented in Table 1 and Table 3.

**Table 1:** Characteristics of the subjects and nutrient intake before incremental test (*n* = 10)

Variables	Mean	SD
Age (years)	23.3	3.6
Total Body Mass (kg)	84.4	9.1
Height (cm)	179.0	0.1
Body Mass Index (kg/m²)	25.6	3.2
Fat Mass (%)	21.8	4.2
Fat Mass (kg)	18.0	4.5
Fat Free Mass (%)	78.1	4.2
Fat Free Mass (kg)	63.4	6.1
Abdomen Perimeter (cm)	88.9	6.5
Hip Perimeter (cm)	99.5	6.5
${\dot{\text{V}}\text{O}}_{2\max}$ (ml/kg/min)	42.8	3.2

Note: ${\dot{\text{V}}\text{O}}_{2\max}$ , Maximum Oxygen Uptake.

**Table 2:** Experimental meals composition

Variables	High Carbohydrate Meal	High-Fat Meal
Energy (kcal)	385.0	382.2
Amount (g)	387.0	223.0
Protein (g)	12.4	8.4
Carbohydrate (g)	70.1	19.6
Fat (g)	6.0	29.9
Monounsaturated Fatty Acids (g)	0.6	12.9
Polyunsaturated Fatty Acids (g)	0.8	7.0
Saturated Fatty Acids (g)	1.5	7.5
Fiber (g)	6.8	5.2
Glycemic Index	48.6	34.3

**Table 3:** Energy and nutrient intake before an incremental test in untrained men (*n* = 10)

Variables		High Carbohydrate Meal		High-Fat meal	
		Mean	SD	Mean	SD
TEI (kcal)		2200.8	556.6	2119.6	456.0
Protein					
	% TEI	24.9	6.3	23.1	6.9
	Kcal	532.2	122.7	483.4	165.6
	g	133.0	30.6	120.8	41.4
Carbohydrate					
	% TEI	43.0	10.2	46.1	5.9
	Kcal	958.8	344.2	991.4	281.9
	g	239.7	86.0	247.8	70.4
Fat					
	% TEI	32.0	9.1	30.6	6.6
	Kcal	703.7	290.9	644.6	193.4
	g	78.8	32.3	71.6	21.4

Note: TEI, total energy intake.

According to Supplementary Table 2, both experimental meals (HCM; HFM, respectively) are isoenergetic (385 kcal; 382 kcal), also presenting similar values of protein (12.4 g; 8.4 g) and fiber (6.8 g; 5.2g). HCM has approximately 72.8% of the calories in the form of carbohydrates (70.1 g) and 14.2% in the form of fat (6.0 g). HFM has 20.5% of calories as carbohydrates (19.6 g) and 70.6% as fat (29.9 g). The absolute amount of fat types presented great variation due to the diametrically different composition of the experimental meals. For HCM, monounsaturated, polyunsaturated, and saturated fatty acids were 0.6 g, 0.8 g, and 1.5 g, respectively. For HFM were 12.9 g; 7.0 g and 7.5 g.

**Figure 2:**
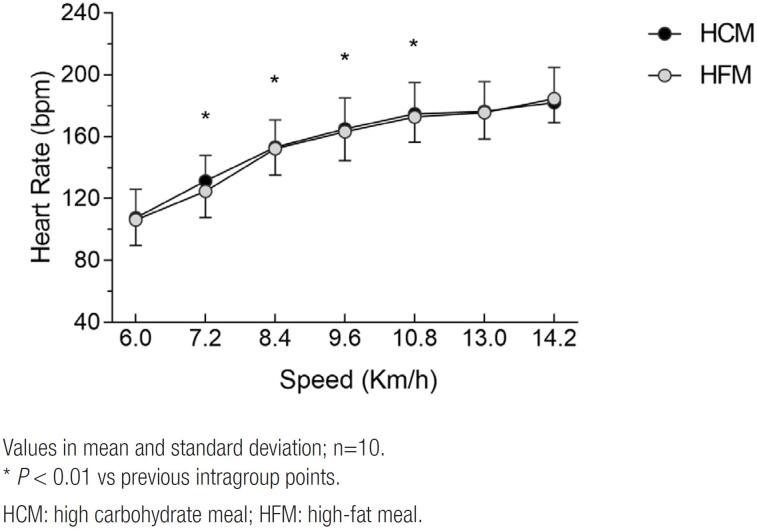
Heart Rate curve over incremental test in different experimental situations.

The Heart Rate was not different between the high carbohydrate and high-fat meals, although it increased according to the intensity of the test over time (*P* < 0.01), however at higher intensities no difference can be observed (*P >* 0.05) (Figure 2).

### High fat meal decreases RER at low intensities

**Figure 3:**
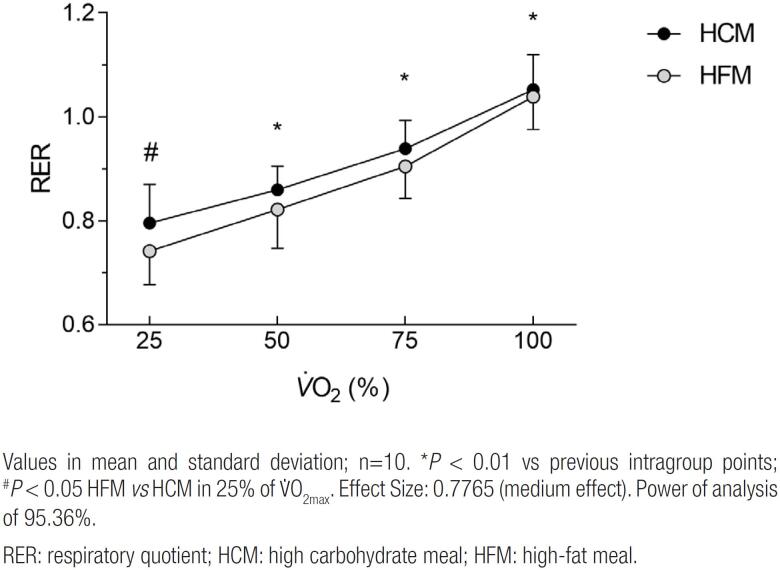
RER behavior curve over incremental test in different experimental situations.

Regarding the behavior of the respiratory coefficient in the comparison of the intragroup points, similar to the carbohydrate oxidation curve and heart rate, an increase was observed throughout the test (*P* < 0.01) (Figure 3). In the comparison between the protocols, however, the RER in the HFM was lower than in the HCM (*P* < 0.05) when compared at the 25% point of ${\dot{\text{V}}\text{O}}_{2\max}$ . The same behavior was not observed at intensities of 50, 75, and 100% (Figure 2).

### Total fat oxidation increases with a high-fat meal during maximal exercise test

**Figure 4:**
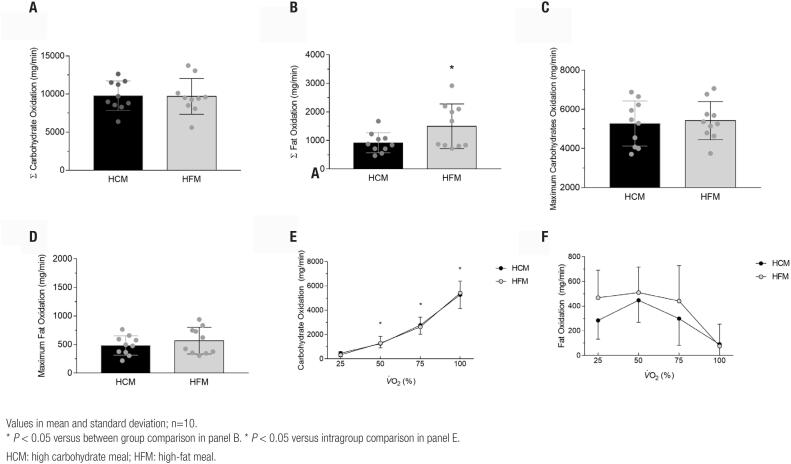
Absolute oxidation, point of maximum oxidation and curve of oxidation of energetic substrates during incremental test. (**A**) Absolute carbohydrate oxidation rate during the different experimental situations. (**B**) Absolute fat oxidation during the different experimental situations. (**C**) Maximum carbohydrate oxidation points for each individual during the different experimental situations. (**D**) Point of maximum fat oxidation for each individual during the different experimental situations. (**E**) Carbohydrate oxidation curve during the different experimental situations. (**F**) Fat oxidation curve during the different experimental situations.

Concerning the absolute oxidation of substrates, the absolute oxidation of carbohydrate showed no difference (*P* > 0.05) between the experimental protocols (Figure 4A). Interestingly, higher oxidation of fat (Figure 4B) was observed in the HFM protocol compared to HCM (*P* < 0.05).

### Substrate oxidation curves and maximum oxidation points do not undergo pre-exercise feeding changes

The point of maximum oxidation of carbohydrates (Figure 4C and 4D) and fat showed no difference when compared with different meal compositions (*P* > 0.05). The fat oxidation curve showed no difference between the protocols nor between the intragroup points. However, the carbohydrate oxidation curve, although it does not show any difference when compared to the two experimental situations, an increase during the test can be observed over time (*P* < 0.01) (Figure 4E and 4F).

## DISCUSSION

This is the first study to observe the acute influence of a high-fat meal on the oxidation of energetic substrates during an incremental exercise test in untrained individuals. It was observed, in addition to a reduction in the RER at low intensities, higher total fat oxidation when compared to a high-fat meal versus a high carbohydrate meal. Although other studies were addressing the pre-exercise feeding, there is usually a comparison between a high carbohydrate meal and fasting, but not with a high-fat meal.

The respiratory quotient showed a difference only at 25% ${\dot{\text{V}}\text{O}}_{2\max}$ intensity between experimental situations, which was also demonstrated in a study that observed lower RER at low intensities when compared to a single glucose meal versus fasting condition (11). However, other authors show maintenance in the RER difference through higher intensities, reaching up to 68% ${\dot{\text{V}}\text{O}}_{2\max}$ with a difference between the respiratory quotients in carbohydrate versus fasting condition. Our sample, when receiving a high fat meal, shows RER equal to the high carbohydrate protocol before 50% ${\dot{\text{V}}\text{O}}_{2\max}$ . Therefore, when compared a fasting protocol with a glucose supply, RER remains low for longer (21,22).

In our investigation, untrained individuals who receive a pre-test high-fat meal exhibit a greater absolute fat oxidation throughout the exercise. Other studies demonstrate similar behavior in total fat oxidation when comparing a fasting protocol with a carbohydrate meal. The same studies cite an increase in total carbohydrate oxidation with the administration of such a meal, while our data suggest no difference in this aspect when comparing HFM and HCM (23). We believe that this can be explained by the training status and the feeding state promoted by both HFM and HCM, compared to fasting (11,21,23,24). Sherman and cols. (22), who compared fasting with a meal with 2.2 g of carbohydrate/kg body weight, also showed no difference in total carbohydrate oxidation during exercise, corroborating our results.

Although the present study did not evaluate the plasma concentration of free fatty acids, the above-mentioned studies did, noting that a carbohydrate meal, at minimum one hour before the exercise, reduces its concentration when compared to the fasting state (22). This may be a determining factor in the difference in total oxidized fat when comparing HCM and HFM. When administered a glucose drink, at lower intensities, subjects begin to substitute the energy protagonism from fat to glycolytic pathway even through decreasing the transport of long-chain fatty acids to the mitochondria via inhibition of Carnitine Palmitoyl Transferase (CPT) (11,25).

The present study found no relationship between the composition of the pre-exercise meals and the point of maximum fat oxidation (MFO), nor the rate of fat oxidation over the test time. When comparing carbohydrate meals with fasting conditions, there is a drop in both the MFO and the fat oxidation rates during the test (11,26).

It should be noted that the individuals of the cited studies are at least moderately trained, which may explain the metabolic behavior of these substrates. Our investigation used untrained subjects, presenting approximately 32% below ${\dot{\text{V}}\text{O}}_{2\max}$ (present study: 42.9 ± 3.3 mL/kg/min versus other studies: 63.0 ± 2.1 mL/kg/min) (11,21,23,24). Physical training generates adaptations in the skeletal muscle, making the individuals more competent in oxidizing fat (5). Mitochondrial biogenesis (2), increase in the amount of fatty acid-binding proteins in skeletal muscle and mitochondria (3), and an improvement in the ATP resynthesis capacity through an increase in the number of proteins involved in the electron transport chain are examples of adaptations linked to the chronic practice of physical exercise (1).

Participants without the aforementioned physiological adaptations submitted to HFM, despite having a more readily available fat substrate for oxidation during exercise, are less competent for fat oxidation, as observed in MFO and fat oxidation rate throughout the exercise. The absolute oxidation of fat can be explained by the fact that, despite not having enough machinery to rapidly oxidize the lipid substrate, the individual will be exposed to this circulating nutrient for a longer time, making use according to their metabolic capacity.

In conclusion, our investigation concludes that, although there is greater absolute oxidation of fat in individuals that consume pre-exercise HFM, this oxidation is more closely related to the availability of the nutrient than necessarily to mobilization competence and main oxidation by the individual. This behavior can be observed at lower intensities of exercise (i.e. 25% of ${\dot{\text{V}}\text{O}}_{2\max}$ ). In practice, to achieve greater peaks of fat oxidation and maintenance of these throughout the exercise, physical training becomes a more powerful and effective ally than the nutritional composition of the pre-exercise meal.
